# Lessons from bacteriophages part 2: A saga of scientific breakthroughs and prospects for their use in human health

**DOI:** 10.1371/journal.ppat.1006970

**Published:** 2018-05-17

**Authors:** Kunica Asija, Carolyn M. Teschke

**Affiliations:** Department of Molecular and Cell Biology, University of Connecticut, Storrs, Connecticut, United States of America; University of Kentucky, UNITED STATES

## Introduction

Bacteriophages, or phages, are considered the most abundant entities on the planet, with their total numbers ranging from 10^30^–10^31^ particles [1]. Phages are known to play an important role in driving the evolution of their bacterial hosts, mostly via generalized and specialized transduction mechanisms [2]. Phage therapy as a means to eliminate bacterial pathogens was used first in the 1920s; however, its use declined due to the advent of antibiotics in the 1940s [3]. Eastern European countries have performed human trials for phage therapy for a century now, including a phage therapy trial in 1938 at the Eliava Institute of Bacteriophage in Georgia that successfully eliminated bacterial dysentery in 74% of the 219 cases by using a phage cocktail that targeted a wide range of causative agents for dysentery [4]. Here, we present some of the major contributions of phage research to human health.

### Phages and health

**Fig 1:**
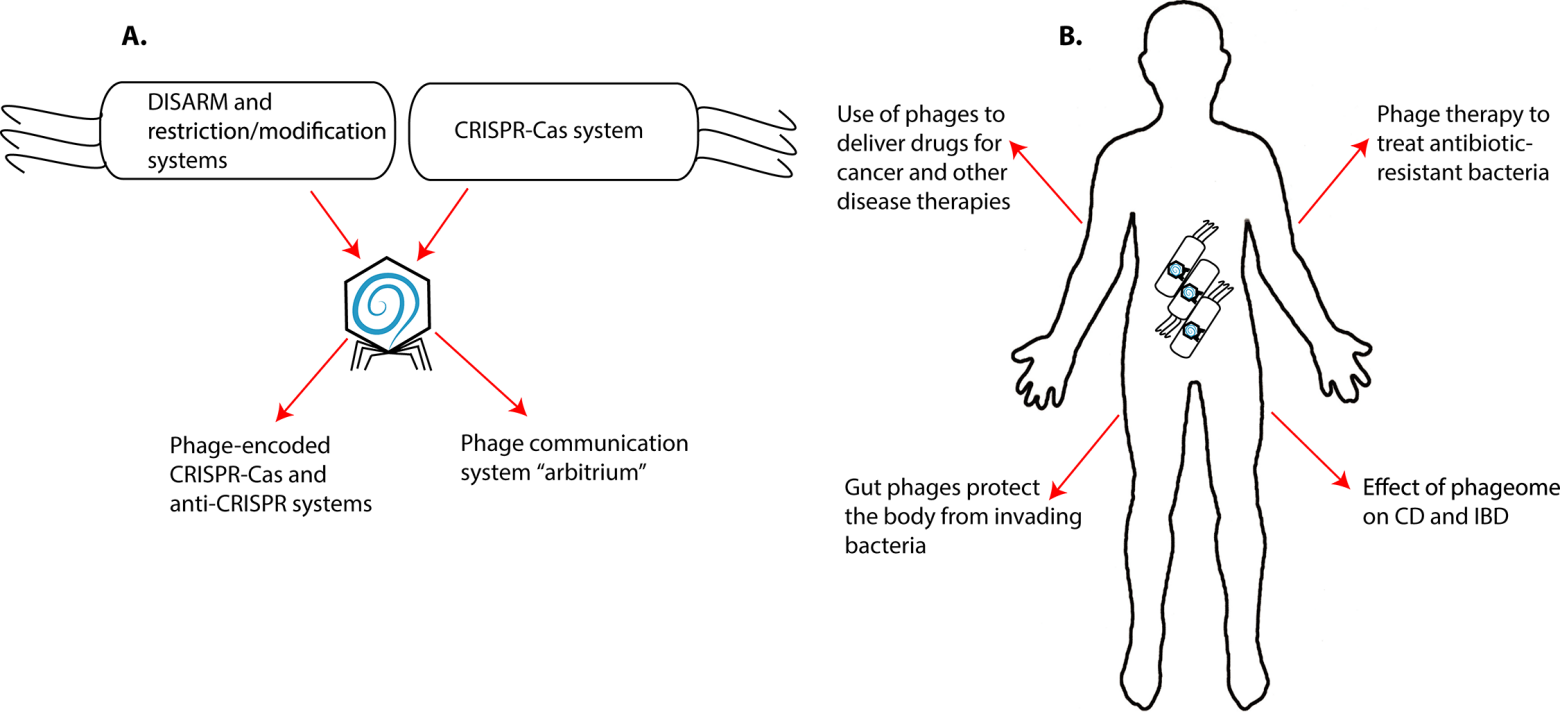
(A) Coevolution of phage with bacterial hosts. Mechanisms of coevolution by phages and their bacterial hosts. (B) Effects of phages on human health. Phages can affect human health directly or can be used after genetic manipulation to affect disease outcomes. Cas, CRISPR-associated protein; CD, Crohn disease; CRISPR, clustered regularly interspaced short palindromic repeat; DISARM, defense island system associated with restriction-modification; IBD, irritable bowel disorder.

The role of the “phageome” or the “virome” in human health is the most recent and could be considered phages’ most significant contribution to human health [5] (Fig 1). In the normal gut, phages reside in mucus and protect the gut from invading bacteria, which need to get through the mucus to invade host cells [6]. However, when a disease leads to gut pathogenesis, this protection can be lost. For example, there is a decrease in the variety of the Caudovirales (tailed) bacteriophages (predominant in the human gut) in Crohn disease (CD) cases compared to the controls, suggesting that the phages likely play a role in the development of the disease [7]. The role of phage dysbiosis is being studied in irritable bowel disorder (IBD) as well. In IBD, the trend observed in Caudovirales bacteriophage richness is inversed to the one seen in the case of CD [8]. The role of phages in exacerbating intestinal inflammation, the primary symptom of IBD, can be in part explained by the release of the phages from their host bacteria in the gut. They can act as antigens and induce the activation of the humoral immune system, thereby aggravating the inflamed state [9].

The role of the phageome in the enrichment of microbial antibiotic resistance genes cannot be overlooked. The increase in antibiotic resistance is attributed to an increase in specific inactivators of drugs such as chloramphenicol acetyltransferase and/or an increase in the multidrug resistance transporters such as the protein MdtK [10]. Following antibiotic treatment, there is in an increase in exchange of genetic material between host microbes and their prophages, bringing about enrichment of genes that almost guarantees the survival of the bacterial host [10].

### Phage therapy for treatment of bacterial infections

In precision medicine, manipulation of an individual’s microbiome has been proposed as a way to treat specific conditions [11]. One could consider the use of the phageome as another treatment, especially to control the epidemic of antibiotic-resistant bacteria. Tailed bacteriophages are most commonly used for phage therapy. One of the main advantages of using phages as antimicrobials over antibiotics is their high rate of replication and that they will continue to replicate as long as there are viable bacterial hosts. Also, the impact they have on the environment compared to antibiotics is minimal since they are largely made of proteins and nucleic acids. Of course, bacteria can become resistant to phages, so a cocktail of different phages is commonly used in phage therapy, and the composition of the cocktail varied as treatment progresses [12].

Some of the success stories of phage therapy include using phage cocktails to treat *Clostridium difficile* infections [13]. The difficulty in treating the *C*. *difficile* infections arises from their naturally antibiotic-resistant biofilms, but phages are able to penetrate into biofilms and kill the microbes. In another study, a combination of antibiotic and phage therapy treated a urinary tract infection caused by a refractory strain of *Pseudomonas aeruginosa*. A two-log reduction in the counts of the organism was seen over a span of 10 days, with a concomitant decline in the bacteriophage counts [14]. This study shows the effectiveness, as well as the safety, of phage therapy.

Multidrug-resistant (MDR) strains of *Acinetobacter baumanii* are an increasing problem for hospitals. In a recent high-profile case, a patient with MDR *A*. *baumanii* was successfully treated with several rounds of phage therapy after all efforts at antibiotic treatments failed to resolve the patient’s infection [15]. Further research for phage therapy to fight MDR *A*. *buamanii* and other MDR bacteria is ongoing [16]. A more advanced application of phage therapy is the use of filamentous phages, such as M13, in targeting cancer cells. The M13 phage has been engineered to selectively target and deliver drugs into prostate cancer cells in in vitro conditions [17].

One of the major risks of using phages for therapy is toxin release from the host cells upon being lysed by phages. Often times, the toxin gene (such as the Shiga toxin gene) is present in the genome of temperate phages and could potentially be expressed during infection of host cells. Another drawback of using phage therapy is the risk of release of lipopolysaccharide (LPS) from gram-negative host cells as a result of phage-induced cell lysis. This is of acute clinical significance since the released LPS or endotoxin can cause heightened inflammation and tissue damage in patients suffering from septicemia [18]. With time, the hope is to use phages routinely for treatment of bacterial infections, eliminating or as a supplement to the use of antibiotics. In the interim, phages are already being used to treat biofilms on medical devices and industrial applications [19].

### The evolutionary arms race

Host cells and their phages constantly coevolve to gain a fitness advantage over each other. As an example, the clustered regularly interspaced short palindromic repeats (CRISPR)–CRISPR-associated protein 9 (Cas9) system is a type-II bacterial adaptive immune system that was discovered in *Streptococcus pyogenes* [20]. CRISPRs are found in many bacterial species as a defense mechanism against bacteriophages and other foreign DNA. A double-stranded (dsDNA) nuclease (e.g., Cas9) is associated with this system and is responsible for the introduction of chromosomal breaks. A CRISPR-associated guide RNA (crRNA) identifies the target DNA sequence that is then acted upon by the Cas9. The ability to cut DNA at specific sites directed by the CRISPR guide RNAs has revolutionized molecular biology and will allow humankind to manipulate genomes at will.

The discovery of anti-CRISPR proteins, which are carried by some phages to evade their host’s CRISPR restriction system, suggests we have not yet found all the tricks phages are able to employ for survival [21]. Phages can even communicate the state of the infection with small molecules. In this communication system, termed “arbitrium,” the progeny phages estimate the number of previous infections by measuring the concentration of the peptides in the medium. Phages belonging to the spBeta group—which includes phages phi3T, phi29, and spBeta—carry an operon that codes for genes to synthesize a short peptide with the amino acid sequence Ser-Ala-Ile-Arg-Gly-Ala (SAIRGA), as well as an intracellular receptor for the peptide and an inhibitor of lysogeny. These peptides are secreted into the medium following the infection of host cells by phages and subsequently transported into bacterial cells via the oligopeptide permease transporter. An increased intracellular concentration of the peptide signals to the phage a switch from lysis to lysogeny. This means that the greater the concentration of the peptide, the more likely the phages are to enter the lysogenic state, allowing the host cells to continue to grow rather than lysing all of the host cells. [22].

Beyond the CRISPR–Cas systems and the well-known restriction modification systems, bacteria have many other antiviral schemes. These include the sirtuins, which are evolutionarily conserved nicotinamide adenine dinucleotide (NAD+)–dependent enzymes shown to restrict viral replication in both mammalian and bacterial cells. They have a range of activities, including adenosine diphosphate (ADP) ribosylation, lysine deacetylation, and also acting as lipoamidases [23]. For example, the CobB protein of *E*. *coli* is a lipoamidase and plays a role in antiphage defense. This was determined by knocking out the gene, infecting the cells with T4 phage, and showing the burst size of the phage increased significantly upon deletion of the *cobB* gene [24]. Another recently discovered defense mechanism in *Bacillus paralicheniformis* is the defense island system associated with restriction-modification (DISARM). DISARM comprises five genes located on defense islands that usually contain the genes involved in host defense. This system uses a restriction modification system to inhibit phage replication and methylates the cytosine in the CCWGG motif of its own DNA (where W could be an A or a T) [25]. It differs from previously known mechanisms since it employs more than one protein for modification. The DISARM system also inhibited phages that lacked the motif, thereby demonstrating its complex and yet-to-be-understood mode of action.

The coevolution of the predator–prey relationship between phages and bacterial cells can be additionally exemplified by *Vibrio cholerae*, which carries an extrachromosomal element called phage-inducible chromosomal island-like element (PLE) (Fig 1). PLE prevents infection and spread of the predatory phage ICP1 into other *V*. *cholerae* populations by inducing cell lysis before progeny phages can be produced. In order to overcome this host defense mechanism, phage ICP1 has evolved its own phage-encoded CRISPR–Cas system that obliterates the PLE in the host cells, thereby furthering its propagation in the host cell population [26]. This sheds light on a new mechanism to ablate pathogenic bacteria, although a deeper understanding will be vital in order for us to benefit from it. Nevertheless, this provides yet another example of how investigating the evolutionary arms race between phages and host cells may provide fruitful advances to humankind.

## Conclusion

Phages are the ideal alternatives to antibiotic use; since they are extremely host specific, they are easy to manipulate and are generally safe for human use. Therefore, phage-based research is a logical step toward eradication of antibiotic-resistant microbes. This is true especially since phages offer a multitude of ways to tackle this problem, whether it is through the use of phage therapy or by genome editing. We are convinced that continued studies of bacteriophages will increase our arsenal of molecular tools for all sorts of uses in biology.
